# Pulmonary Actinomycosis in a 65-Year-Old Female with Poor Oral Dentition

**DOI:** 10.3390/diagnostics14131421

**Published:** 2024-07-03

**Authors:** Sha Yi, Rabindra Ghimire, Thomas A. Sporn, Ann T. Sutton, Dora A. Lebron Figueroa, John E. Markantonis

**Affiliations:** 1 Department of Pathology and Laboratory Medicine, Brody School of Medicine, East Carolina University, Greenville, NC 27834, USA; yis22@ecu.edu (S.Y.); spornt20@ecu.edu (T.A.S.); suttonan@ecu.edu (A.T.S.); 2 Department of Medicine, Division of Infectious Diseases, Brody School of Medicine, East Carolina University, Greenville, NC 27834, USA; ghimirer16@ecu.edu (R.G.); lebronfiguerod15@ecu.edu (D.A.L.F.); 3 ECU Health, ECU Physicians-Pathology, Greenville, NC 27834, USA

**Keywords:** actinomyces, actinomycosis, pulmonary actinomycosis, thoracic actinomycosis

## Abstract

Pulmonary actinomycosis is an uncommon clinical entity that can be challenging to diagnose due to its non-specific symptomatology. Misdiagnosis and delayed treatment may result in invasive procedures and extended antimicrobial treatment courses. We report a case involving a 65-year-old female with poor oral dentition admitted for acute respiratory failure subsequently found to have a left-sided pleural effusion and perihepatic abscess formation. Cytopathology examination and microbiology studies confirmed the diagnosis of pulmonary actinomycosis.

A 65-year-old female with hypertension, hyperlipidemia, and peripheral vascular disease presented to the emergency department (ED) of an affiliated hospital with progressive shortness of breath, cough, upper abdominal pain, and subjective fever for two weeks. Additionally, she was seen at the same ED seven weeks prior to this presentation for left arm pain of several weeks duration and was found to have a significantly elevated erythrocyte sedimentation rate (>130 mm/h; reference range, <30 mm/h). This led to an initial diagnosis of polymyalgia rheumatica, for which she had recently completed a tapered course of oral prednisone.

On arrival, she was found to be afebrile but tachycardic (116 beats per minute), tachypneic (26 breaths per minute), and hypoxic (SpO_2_ 60–70% on room air). On physical examination, there was diminished air entry in the right lung and poor oral dentition, visualized with several loose or missing teeth along with visible tooth decay (dental caries). A complete blood count analysis revealed leukocytosis (25.57 k/µL; reference range, 4.50–11.00 k/uL). A comprehensive metabolic panel was unremarkable except for decreased carbon dioxide (14 mEq/L; reference range, 23–31 mEq/L). Elevated lactic acid (9.4 mmol/L; reference range, 0.5–2.0 mmol/L) and procalcitonin (10.05 ng/mL; reference range, 0.00–0.50 ng/mL) levels were detected. Chest X-ray imaging showed near-complete opacification of the right hemithorax. A computed tomography (CT) angiography of the chest revealed a large right hydropneumothorax with a leftward mediastinal shift (Figure 1A). The pleural fluid crossed the diaphragm, terminating in a thick-walled collection along the lateral aspect of the right lobe of the liver (Figure 1B). She developed progressive respiratory failure, requiring intubation and mechanical ventilation. The patient was administered intravenous (IV) vancomycin and piperacillin/tazobactam and was subsequently transferred to our facility for a thoracic surgery evaluation.

The patient underwent a right thoracostomy with a chest tube placement, yielding 1 L of purulent fluid. Her pleural fluid cell count analysis revealed >59 K red blood cells/µL and >45 K nucleated cells/µL with a neutrophilic predominance. Additional analysis of the fluid yielded pH 6.4, protein 4.3 g/dL (serum 6.1 g/dL; reference range, 6.2–8.1 g/dL), and a lactate dehydrogenase level of 9211 U/L (serum 213 U/L; reference range, 125–220 U/L). A cytological examination of the pleural fluid revealed large, intertwined aggregates of filamentous micro-organisms on Gomori methenamine silver staining (Figure 2). The organism was not highlighted by acid-fast staining. The Gram stain of the pleural fluid demonstrated large bacterial clusters (Figure 3A) composed of filamentous, branching Gram-positive bacilli with a beaded appearance (Figure 3B) measuring up to 31 mm in diameter. Notably, additional bacterial morphologies were not seen. The culture of the pleural fluid initially grew 1+ *Pseudomonas aeruginosa* with two morphologies of anaerobic Gram-negative rods identified on the fifth day of plate reading. These isolates were subsequently identified as a *Fusobacterium* species (4+) and *Campylobacter gracilis* (3+). Given that there was no organism isolated with a similar morphology to that seen on the direct Gram stain, the incubation of the primary plates was extended to 14 days. This was based on the clinical determination of the microbiology lab director to isolate the predominant organism seen on direct Gram stain. 

On day 14 of incubation, tiny “spider” type microcolonies were noted on the chocolate agar plate incubated aerobically at 35 °C with 5% CO_2_ [1]. A secondary culture onto another chocolate agar plate and anaerobic incubation at 37 °C produced predominantly white, opaque, smooth, circular colonies as well as colonies with a “molar tooth” appearance (Figure 4, white arrow) [1]. Gram staining of these colonies revealed Gram-positive, beaded rods. A modified Kinyoun stain was negative. Identification of the isolate was attempted by matrix-assisted laser desorption/ionization time of flight mass spectroscopy (MALDI-TOF); however, identification was unsuccessful. The isolate was sent to Mayo Clinic Laboratories (Rochester, MN, USA), where it was identified as *Actinomyces gerencseriae* by 16S rRNA gene sequencing.

A right perihepatic drain was placed to address the thick-walled collection along the lateral aspect of the right liver lobe, with 35 mL of purulent material collected. The patient received four weeks of piperacillin/tazobactam prior to transitioning to amoxicillin-clavulanate. A CT scan of the abdomen performed at the completion of IV therapy revealed near resolution of the perihepatic abscess and a low-volume residual loculated pleural effusion. The perihepatic drain was removed, and the patient continued amoxicillin/clavulanate to complete a six-month total duration of antimicrobial therapy. A subsequent CT scan of the abdomen showed complete resolution of the perihepatic abscess (Figure 5A) and empyema (Figure 5B).

*Actinomyces* was initially discovered in 1877 by the pathologist Otto Bollinger [2,3]. Actinomycosis, a chronic granulomatous infection caused by the *Actinomyces* species, was first described in humans in 1896 [3,4]. Recent phylogenetic studies utilizing 16S rRNA gene sequencing and whole genome sequencing have reclassified many former *Actinomyces* species into several new genera (e.g., *Schaalia*, *Winkia*, etc.) [4,5]. *Actinomyces* species and related genera usually appear as filamentous, branching bacilli that stain Gram-positive with a beaded appearance on direct Gram staining of clinical samples [2,4]. On sub-culture to solid media, this morphology is often lost [4,6]. Most *Actinomyces* species and related genera are non-spore-forming aerotolerant anaerobes, but some are obligate anaerobes [2,6]. Colony morphology and microscopic characteristics can be highly variable in this family of organisms [6]. Isolation of the primary agent in cases of actinomycosis can be challenging due to their slow growth rate and the polymicrobial nature of these infections. *Streptococcus anginosis* group, *Fusobacterium* spp., *Propionibacterium* spp., *Campylobacter gracilis*, anaerobic Gram-positive cocci, and anaerobic Gram-negative rods are often identified as co-pathogens in actinomycosis [3,7]. Extended incubation of cultures may increase the identification yield for *Actinomyces* species and related genera; however, this is not a routine practice in most clinical laboratories [6]. The overall yield upon extension of incubation time is low with an increasing risk of contamination. Thus, the decision to extend incubation time should be carefully determined by the microbiology lab director based on clinical judgment. Due to the low diagnostic yield of culture in these cases, the diagnosis of actinomycosis is generally accomplished by clinical findings and cytohistologic evaluation of specimens [2,3,4].

In addition to microscopic evaluation of specimens and bacterial culturing, macroscopic evaluation of sampled purulent fluid for sulfur granules can aid in the diagnosis of actinomycosis [4]. When sulfur granules are crushed, stained, and viewed under a microscope, the characteristic morphology of *Actinomyces* species and related genera should be present [4]. It is important to note that not all *Actinomyces* species and related genera produce sulfur granules, and some *Nocardia* spp. can produce them as well. Due to differences in therapeutic strategies between these two organisms, it is important to distinguish them [8]. The modified acid-fast bacilli stain is an important tool that can support decision-making. *Nocardia* spp. stain positively, while *Actinomyces* species and related genera are negative [8]. This staining can be performed directly on specimens or on cultured isolates [4,6]. Definitive identification is generally accomplished through MALDI-TOF or sequencing [4,6,9]. These methods have largely replaced biochemical testing due to their ability to provide accurate genus-level identification [4].

The diagnosis of actinomycosis can be challenging for medical professionals, delaying the correct diagnosis in a high proportion of cases. This can result in prolonged antimicrobial courses and the need for invasive procedures. Isolation of *Actinomyces* species and related genera from culture can be challenging; diagnosis is often accomplished by cytohistologic evaluation.

## Figures and Tables

**Figure 1:**
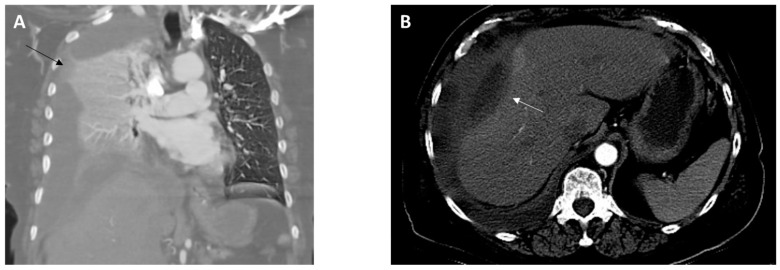
Images from computed tomography angiography of the chest obtained at admission. (**A**), Coronal view revealing a collapsed right lung due to a large pleural effusion (black arrow). (**B**), Axial view showing the presence of a perihepatic abscess (10.0 × 3.8 cm) (white arrow).

**Figure 2:**
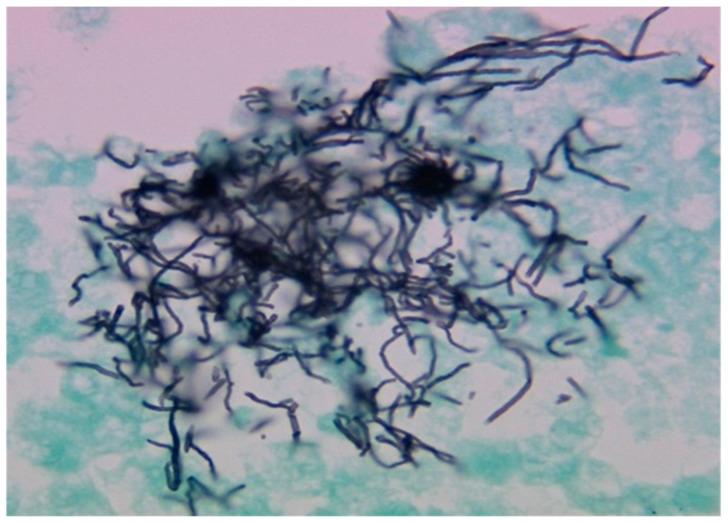
Cytological examination of the pleural fluid revealed large, intertwined aggregates of filamentous, branching bacteria on Gomori methenamine silver stain (1000× original size, oil immersion).

**Figure 3:**
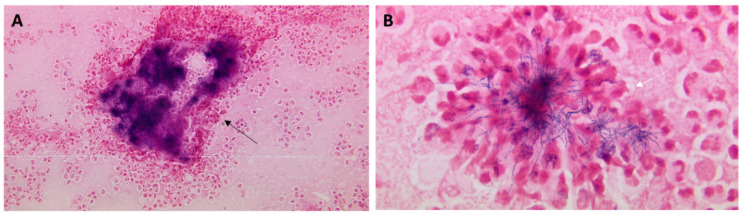
Photographic images of the Gram stain from the pleural fluid culture. (**A**), original magnification, 200×. (**B**), original magnification, 1000×, oil immersion. The Gram stain demonstrated large bacterial clusters of filamentous, branching bacteria surrounded by a dense collection of neutrophils (black arrow). Higher-power magnification (600×) revealed filamentous, branching, gram-positive bacilli with a beaded appearance (white arrow).

**Figure 4:**
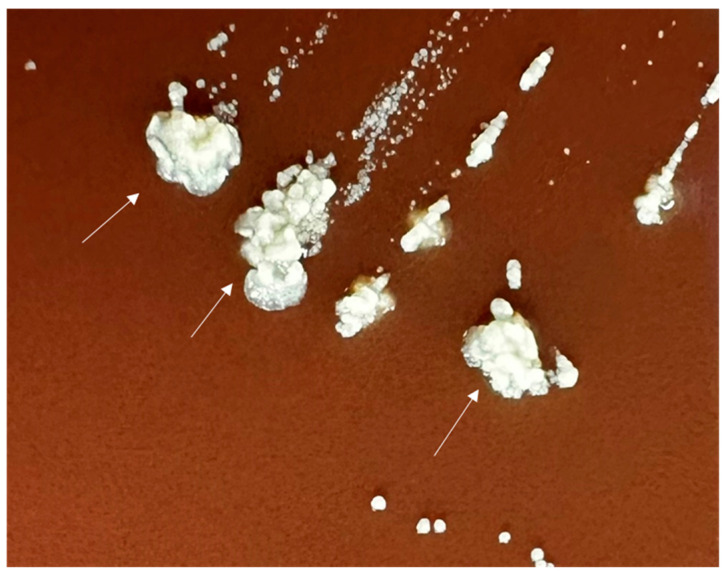
Photographic image of isolated colonies of *Actinomyces gerencseriae* following sub-culture onto a chocolate agar plate and anaerobic incubation at 37 °C for 14 days. The larger, mature colonies are exhibiting the characteristic molar tooth-appearance seen in *Actinomyces gerencseriae* (white arrow).

**Figure 5:**
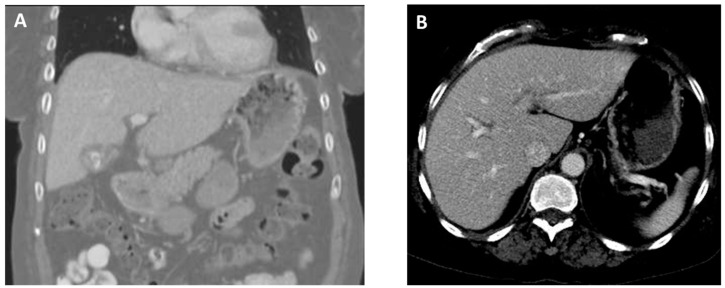
CT abdomen/pelvis with intravenous contrast obtained four months following hospital discharge shows resolution of the empyema ((**A**), coronal view) and perihepatic abscess ((**B**), axial view).

## Data Availability

Data that may be potentially identifiable is not publicly available due to patient confidentiality and privacy considerations. The data presented in this study that are non-identifiable are available on request from the corresponding author.
